# ^13^C-labeled single-cell Raman sorting reveals sulfur-driven dark carbon fixation in coastal sediments

**DOI:** 10.1093/ismeco/ycag073

**Published:** 2026-03-24

**Authors:** Xiao-Lan Yue, Yue-Hong Wu, Dao-Qiong Zheng, Cong Sun, Lin Xu, Li Cui, Xue-Wei Xu

**Affiliations:** School of Oceanography, Shanghai Jiao Tong University, Shanghai 200030, China; State Key Laboratory of Submarine Geoscience, Second Institute of Oceanography, Ministry of Natural Resources, Hangzhou 310012, China; State Key Laboratory of Submarine Geoscience, Second Institute of Oceanography, Ministry of Natural Resources, Hangzhou 310012, China; Ocean College, Zhejiang University, Zhoushan 316062, China; College of Life Sciences and Medicine, Zhejiang Sci-Tech University, Hangzhou 310018, China; College of Life Sciences and Medicine, Zhejiang Sci-Tech University, Hangzhou 310018, China; Key Laboratory of Urban Environment and Health, Institute of Urban Environment, Chinese Academy of Sciences, Xiamen 361021, China; State Key Laboratory of Submarine Geoscience, Second Institute of Oceanography, Ministry of Natural Resources, Hangzhou 310012, China; National Deep Sea Center, Ministry of Natural Resources, Qingdao 266237, China

**Keywords:** single-cell Raman spectroscopy, stable isotope labeling, chemoautotrophs, carbon fixation, coastal sediment, *Guyparkeria*

## Abstract

Chemoautotrophs drive carbon fixation in coastal sediments, but most of them remain uncultured with poorly characterized *in situ* activities. In this study, a cultivation-independent single-cell approach combining Raman spectroscopy with ^13^C-stable isotope probing was developed to enable direct identification of active chemoautotrophs in coastal sediments using function-specific spectral biomarkers, targeted metagenomic sequencing and pure culture verification. ^13^C-induced shifts in cytochrome *c* (749, 1129, 1312, 1589 cm^−1^) and phenylalanine (1002 cm^−1^) Raman bands were systematically evaluated and applied as functional biomarkers through investigations of both representative chemoautotrophic strains and environmental samples. The combined analysis of targeted sorting of active chemoautotrophic cells and metagenomic sequencing revealed dominant species and a complete Calvin-Benson-Bassham (CBB) cycle pathway in sulfur-oxidizing guilds. Remarkably, a novel sulfur-oxidizing chemoautotroph, *Guyparkeria* sp. TX1, which showed ≥99% gene sequence similarity to contigs recovered from sorted-cell metagenomes, was isolated from enrichment cultures. Its significant carbon fixation capacity provided experimental validation for the effectiveness of Raman-based *in situ* functional screening. This study establishes Raman-based functional biomarkers applicable to chemoautotrophic carbon fixation, enabling *in situ* mapping of microbial carbon fluxes. By integrating single-cell phenotypic activity with genomic potential, this work advances the mechanistic understanding of sulfur-driven dark carbon fixation, which sustains coastal blue carbon ecosystems as a keystone process.

## Graphical Abstract

**Graphical Abstract:**
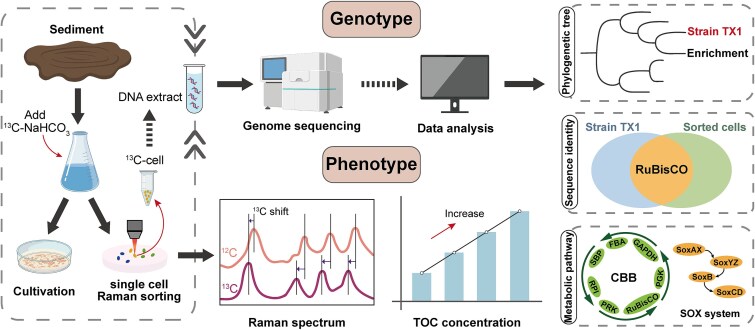

## Introduction

Marine biological carbon fixation driven by photoautotrophs and chemoautotrophs can lock up and sequester organic carbon in biomass, contributing nearly half of global primary productivity and representing a critical component of blue carbon storage [1, 2]. As pivotal contributors to global carbon cycling, coastal ecosystems exhibit carbon fixation rates exceeding 2000 g C·m^−2^·y^−1^ [3–5], with sediments constituting the predominant marine carbon sink via non-photosynthetic dark carbon fixation [6]. This process is primarily mediated by chemoautotrophic communities utilizing the Calvin-Benson-Bassham (CBB) cycle and reductive tricarboxylic acid (rTCA) cycle [7]. Meanwhile, sulfate reduction serves as the primary respiration process in this environment [8], generating a substantial reservoir of reduced sulfur compounds that can fuel chemoautotrophic sulfur oxidation [9]. Consequently, sulfur-oxidizing bacteria, particularly autotrophic *Gammaproteobacteria*, emerge as keystone taxa in sediment carbon cycling [7, 10–12]. Despite their ecological prominence, the mechanistic drivers and microbial actors of dark carbon fixation in coastal sediments remain poorly resolved. Genomic and isotopic approaches have advanced the understanding of carbon-fixing microorganisms and their efficiency in coastal sediments [7, 13, 14]. However, these approaches are largely limited to high-abundance groups, often overlook rare but functionally critical populations, and lack the resolution to link metabolic activity to individual cells, thereby hindering accurate quantification of ecosystem-scale carbon fluxes in dynamic environments.

Single-cell Raman spectroscopy (SCRS) offers a cultivation-independent, activity-based solution to address this challenge [15, 16]. When combined with stable isotope labeling, such as using ^13^C, ^2^H, or ^15^N, specific Raman bands shift to lower wavenumbers due to intracellular assimilation of isotope-labeled substrates. The substitution of light atoms with heavier isotopes during the synthesis of biomolecules serves as an indicator of target metabolic activities [17–20]. Stable isotope labeling with ^13^C does not alter substrate chemistry and is widely regarded as phenotypically neutral in microbial stable isotope probing (SIP) experiments [21]. At present, several researchers have used SCRS with ^13^C stable isotope labeling to study carbon fixation phenotypes of photoautotrophic microorganisms in surface seawater. Shifts in the carotenoid and phycobilin Raman bands have been used to quantify single-cell carbon assimilation and growth rates of photoautotrophic organisms such as cyanobacteria and diatoms [22–26]. Beyond pigment-based biomarkers, subsequent studies demonstrated that shifts of the phenylalanine Raman band provide a reliable and broadly applicable proxy for the incorporation of ^13^C into newly synthesized cellular biomass [27–29]. Quantitative changes in this band have been consistently linked to metabolic activity and cellular growth in a wide range of photoautotrophic, heterotrophic, and environmental microorganisms [30–32]. The further integration of single-cell sorting and targeted metagenomic sequencing contributes to the investigation of taxa and genomic attributes of carbon-fixing bacteria [33]. However, carotenoids which are the accessory pigments ubiquitous in photosynthetic microorganisms are irrelevant to the survival of chemoautotrophic microorganisms [22]. Consequently, the Raman biomarkers developed in previous studies are inapplicable to chemoautotrophs, creating a critical methodological gap in studying non-photosynthetic carbon fixation pathways.

Here, this study bridges this gap by developing a novel single-cell SIP-Raman approach (^13^C-stable isotope probing coupled with Raman spectroscopy) tailored for chemoautotrophic communities in coastal sediments. Cytochrome *c* (Cyt *c*) and phenylalanine Raman band shifts were identified and validated as functional biomarkers of intracellular ^13^C incorporation and metabolic activity in chemoautotrophs. The reliability of these biomarkers was further validated by coupling SCRS with single-cell sorting and targeted genome sequencing. Furthermore, a novel *Guyparkeria* sp. TX1 strain dominant in the coastal sediment enrichment cultures was isolated successfully, and its carbon fixation capacity and genomic machinery for sulfur-driven autotrophy was determined. By integrating phenotypic screening with genome-resolved metagenomics, this study advances the mechanistic understanding of chemoautotroph-mediated carbon cycling and establishes a new framework for probing microbial functions in natural environments.

## Materials and Methods

### Collection of the coastal sediment

Coastal sediment sample was collected from the nearshore mudflat in Zhoushan, PR China (122°24′E; 29°56′ N) at a depth of ~20 cm. The samples were immediately placed in cooler upon collection and stored at 4°C prior to use after being transported back to the laboratory.

### Sample preparation

The chemoautotrophic representative strains *Thiobacimonas profunda* CGMCC 1.12377^T^ [34], *Halothiobacillus diazotrophicus* GDMCC 1.4095^T^ [35] and *Thiomicrorhabdus indica* MCCC 1A13986^T^ [36] was obtained from the China General Microbiological Culture Collection Center (CGMCC), Guangdong Microbial Culture Collection Center (GDMCC) and Marine Culture Collection of China (MCCC), respectively.

Chemoautotrophic reference strains were cultivated under conditions described in previous studies [34–36]. After pre-cultivation, 400 μL of the bacterial solution were added in a 40 ml serum bottle filled with 20 ml of artificial seawater (ASW) medium. After sealing, the headspace gas was replaced with a mixture of N_2_ and O_2_ (v/v, 4/1) to eliminate interference from atmospheric CO_2_. Three experimental groups were prepared: (i) addition of ^13^C-NaHCO_3_ (99 atom%, 99% purity, Aladdin, China), (ii) addition of ^12^C-NaHCO_3_ (Sangon, Shanghai, PR China), and (iii) no additional carbon source. The final concentration of the added ^12^C and ^13^C carbon sources was 2 mM. All three groups were incubated for 3 days under dark conditions, and triplicate incubations were performed for each group.

Approximately 2 g of coastal sediment sample was incubated in ASW medium supplemented with 2 mM ^13^C-labeled bicarbonate under conditions identical to those described above. After 5 days of incubation, microbial cells were extracted using Nycodenz density gradient separation as previously described [18]. Detailed process can be found in Supplementary Information (SI 1).

### Raman spectroscopy and single-cell sorting

SCRS were acquired using a LabRAM Aramis (HORIBA Jobin-Yvon) confocal micro-Raman system equipped with a 300 g/mm grating, 532 nm Nd:YAG laser, and a 100× dry objective with a numerical aperture of 0.9 (Olympus, Japan), providing a spectral resolution of ~3 cm^−1^. The spectra were collected in the range of 500–3200 cm^−1^ with an acquisition time of 5 s and a laser power of 10 mW. Approximately 50 spectra were obtained and averaged for each sample. Spectral preprocessing included baseline correction, averaging, and normalization. Subsequent analyses focused on the 500–1800 cm^−1^ fingerprint region.

Raman spectra from ^12^C-labeled control samples were used as references, and cells exhibiting reproducible isotopic Raman band shifts under ^13^C labeling were identified as actively incorporating ^13^C. Ten cells, identified as ^13^C -metabolizing (with ^13^C Raman band shifts), were sorted using a pulse laser ejection system (PRECI SCI, HOOKE Instruments Ltd., Changchun, PR China) into a receiver containing 3 μL of PBS, as described by Song et al. [20]. Due to the low DNA concentration in the sorted cells, multiple displacement amplification (MDA) was performed using the REPLI-g Single Cell Kit (QIAGEN, Germany), as described by Rinke et al. [37].

### Metagenomic sequencing of sorted cells and the sediment sample

The MDA products of sorted cells were sent to Novogene Bioinformatics Technology Corporation (Beijing, PR China) for sequencing on the Illumina Hiseq 2500 platform using the PE150 strategy. Raw reads were quality-filtered, assembled, and annotated using established pipelines for single-cell genomics and metagenomic analysis [38–41]. The abundances of contigs were calculated using BWA version 0.7.17 [42], SAMtools version 1.9 [43], and BEDTools version 2.26.0 [44].

Metagenomic sequencing of sediment samples and enrichment cultures was performed at Magigene Technology Corporation (Guangdong, PR China). Sequence assembly, binning, refinement, and quality assessment of metagenome-assembled genomes (MAGs) followed standard workflows using MetaWRAP software version 1.2.1 [45]. Only MAGs with completeness >50% and contamination <10% were retained for downstream analyses. Taxonomic classification and phylogenomic reconstruction were performed using the Genome Taxonomy Database framework [46].

Detailed bioinformatic parameters are provided in SI 2.

### Isolation, cultivation, and genome sequencing of the carbon-fixing strain

After enrichment, ~0.1 g of the enriched sediment was suspended in sterile culture medium and diluted 1000-fold to obtain a homogeneous suspension. Approximately 100 μL of the diluted suspension was then spread onto agar plates containing organic carbon-free medium (SI 4) to isolate and purify carbon-fixing bacteria. After incubation, colonies were transferred into liquid medium of identical composition for further purification. A single colony, designated strain TX1, was obtained after repeated subculturing and preserved for subsequent analyses. Moreover, this strain was also deposited into the China General Microbiological Culture Collection Center (CGMCC) under the deposition number of CGMCC 1.65354.

Genomic DNA was extracted from pure cultures using the Magnetic Soil and Stool DNA Kit (TIANGEN Biotech, Beijing, China) according to the manufacturer’s instructions and sent to Magigene Technology Corporation for whole-genome sequencing, with the genomic assembly and quality estimation performed as described above [45]. Targeted metagenomic read mapping and downstream processing were performed using standard pipelines. Detailed procedures and parameters are provided in SI 3.

### Determination of carbon fixation efficiency of strain TX1

The isolated strain TX1 was cultured with DSMZ medium 574 [47]. Since no additional organic carbon sources were introduced into the medium other than the inoculated strain TX1, the increase in total organic carbon (TOC) after cultivation can be considered the amount of net carbon fixation by the strain. Therefore, the TOC concentration in the culture can serve as an indicator for evaluating carbon fixation efficiency [48]. The collected sample was filtered through a 0.7 μm glass microfiber membrane (GF/F, Whatman, China) [49–51]. TOC analysis was performed using a TOC analyzer (TOC-LCPH-SSM5000, Japan) to assess microbial carbon fixation efficiency. Prior to TOC analysis, samples were acidified to pH 4.0 to remove inorganic carbon by converting it to dissolved CO_2_, thereby ensuring that only organic carbon was quantified.

## Results

### Determination of chemoautotrophic Raman signatures using ^13^C labeling

To identify the Raman signals of chemoautotrophic bacteria, the representative strains *Thiobacimonas profunda* CGMCC 1.12377^T^, *Halothiobacillus diazotrophicus* GDMCC 1.4095 ^T^, and *Thiomicrorhabdus indica* MCCC 1A13986^T^ were selected for SCRS in this study. These strains exhibited characteristic Raman bands almost identical to pure Cyt *c* at 747 cm^−1^ (pyrorole breathing), 1125 cm^−1^ [ν(C-N)], 1312 cm^−1^ [δ(C-H)], and 1584 cm^−1^ [ν(C-C)] as reported by Okada et al. [52]. The chemical bonds attributed to the four signature Raman bands of Cyt *c* are all carbon-containing, providing a potential target for ^13^C stable isotope incorporation during carbon fixation. Therefore, it was hypothesized that shifts in the Cyt *c* Raman bands could serve as evidence of chemoautotrophic activity.

**Figure 1:**
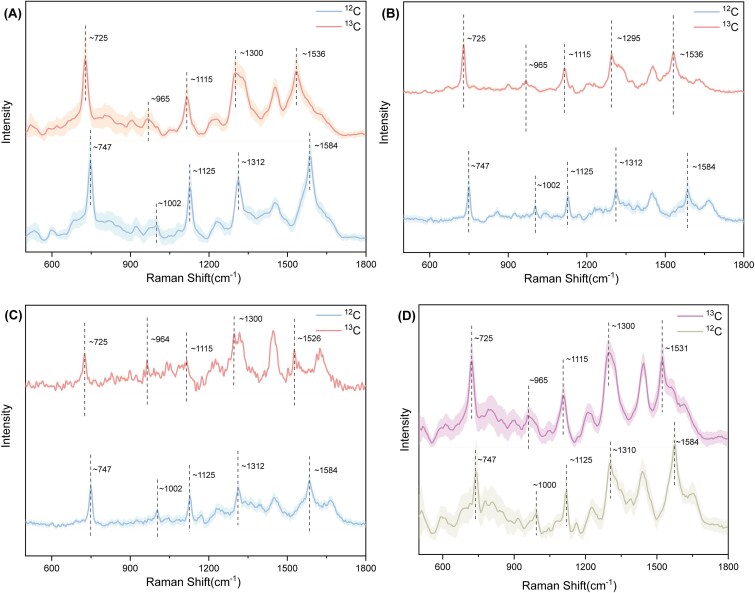
(A) Mean Raman spectra of *Thiobacimonas profunda* incubated 3 days with ^13^C-NaHCO_3_ (red) and ^12^C-NaHCO_3_(blue). (B) Mean Raman spectra of *Halothiobacillus diazotrophicus* incubated 3 days with ^13^C-NaHCO_3_ (red) and ^12^C-NaHCO_3_(blue). (C) Mean Raman spectra of *Thiomicrorhabdus indica* incubated 3 days with ^13^C-NaHCO_3_ (red) and ^12^C-NaHCO_3_(blue). (D) Mean Raman spectra of enrichment cells incubated 5 days with ^13^C-NaHCO_3_ (pink) and ^12^C-NaHCO_3_ (celadon). Shaded areas represent the standard deviation of the mean spectra derived from multiple single-cell measurements.

The average Raman spectra of respective 50 individual cells under two different carbon source conditions showed that after incubation with ^13^C-NaHCO_3_, the Cyt *c* Raman bands at 747, 1125, 1312, and 1584 cm^−1^ were markedly shifted to 725, 1115, 1300, and 1536 cm^−1^, respectively (Figs 1A–C), which confirmed the substitution of ^12^C with the heavier ^13^C, resulting into a decrease in the vibration frequency of C-N, C-H, or C=C bonds. The strong Raman signals from Cyt *c* in the spectra of the chemoautotrophic strains, along with the observed bands shift, indicate that ^13^C was assimilated by the cells. Consequently, the simultaneous shifts of four Raman bands at 747, 1125, 1312, and 1584 cm^−1^ in Cyt *c* were used as functional criteria to identify and sort chemoautotrophic bacteria assimilating ^13^C-NaHCO_3_. Additionally, the Raman band at 1002 cm^−1^, corresponding to phenylalanine, shifted toward 965 cm^−1^ after incubation with ^13^C-labeled substrate. Given that the 3-day incubation may not have been sufficient to achieve full isotopic equilibrium, intermediate peaks between 965 and 1002 cm^−1^ were also observed, consistent with partial ^13^C incorporation.

### Targeted sorting and metagenomic validation of chemoautotrophic cells from coastal sediment

Based on characteristically chemoautotrophic Raman spectra using ^13^C labeling, chemoautotrophic bacteria in the enriched sample of coastal sediment were identified. After incubation with artificial seawater amended with 2 mM ^12^C- or ^13^C-NaHCO_3_, bacteria were isolated by gradient density centrifugation. Raman spectra of carbon-fixing bacteria in the enriched samples of coastal sediment under different carbon source conditions were detected (Fig. 1D). Although the characteristic Raman bands of Cyt *c* in the sediment samples differed slightly from those of pure Cyt *c*, they were similarly red-shifted when the cells assimilated ^13^C-NaHCO_3_, shifting from the initial positions of 747, 1125, 1310, and 1584 cm^−1^ to 725, 1115, 1300, and 1531 cm^−1^, respectively. Additionally, the phenylalanine band shifted from 1000 cm^−1^ to 965 cm^−1^, consistent with the shifts observed in *Thiobacimonas profunda* CGMCC 1.12377^T^.

SCRS permits the non-destructive detection of targeted cells, and the subsequent combination of single-cell sorting and targeted metagenomic sequencing can provide a robust method for linking phenotypes to genotypes. In this study, cells exhibiting ^13^C-induced Cyt *c* and phenylalanine Raman band shifts were sorted from samples of coastal sediment incubated with ^13^C-NaHCO_3_ based on Raman sorting criteria using single-cell laser ejection (Fig. S1). A total of 7 groups were selected, with each group consisting of 10 cells. All cells exhibited significant Cyt *c* and phenylalanine Raman band shifts. The cells obtained from the sorting were then subjected to targeted metagenomic analysis and identified using 16S rRNA gene amplification and cloning to verify the success of MDA, and 5 groups were successfully amplified. The results revealed that four chemoautotrophic Raman biomarker clones belonged to genus *Guyparkeria*, and two clones belonged to genus *Pseudomonas*. Meanwhile, both of the enrichment and sediment samples were subjected to metagenomic sequencing for comparative analysis.

**Figure 2:**
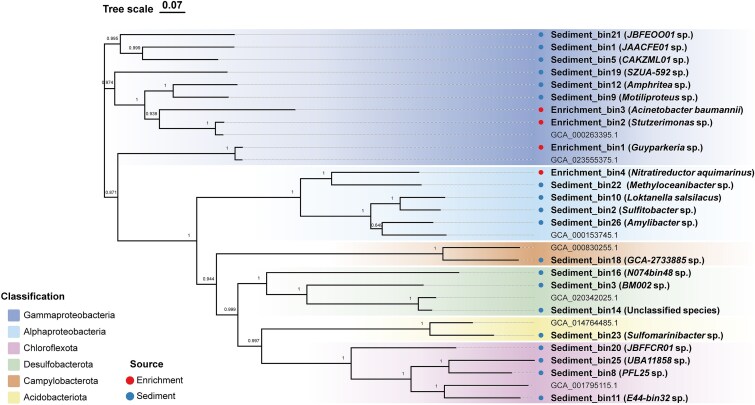
The maximum likelihood phylogenomic tree of bacterial MAGs generated in GTDB-Tk. Bootstrap support values are shown as text at the corresponding nodes. Red and blue points indicated MAGs derived from enrichment and sediment, respectively.

A total of 13.7 Gb of raw sequencing data were obtained from the sediment metagenome, 7.6 Gb from the enrichment metagenome, and 7.8 Gb from the targeted sorted-cell metagenome. Contigs of the targeted metagenomes of the sorted cells were assigned to specific taxa based on sequence similarity, revealing that approximately half of the sorted cells were *Gammaproteobacteria*, and the remaining dominant taxa included *Alphaproteobacteria, Betaproteobacteria*, and *Bacillota*. A total of 4 and 23 medium- and high-quality MAGs were binned from the metagenomic sequencing of enrichment and sediment samples respectively, and the detailed MAG information was listed (Table S1). Based on the phylogenomic reconstruction of those MAGs, four enrichment-derived MAGs were classified into the classes *Alphaproteobacteria* (n = 1) and *Gammaproteobacteria* (n = 3; Fig. 2). While the sediment-derived MAGs taxonomically belonged to the phyla *Acidobacteriota* (n = 1), *Campylobacterota* (n = 1), *Chloroflexota* (n = 4), *Desulfobacterota* (n = 3) and *Pseudomonadota* (n = 10). AAI calculations and phylogenomic analysis showed that enrichment-derived MAGs were phylogenetically distinct from sediment-derived MAGs. This suggests that these chemoautotrophic bacteria were not dominant in the original sediment but were selectively enriched under laboratory conditions.

**Figure 3:**
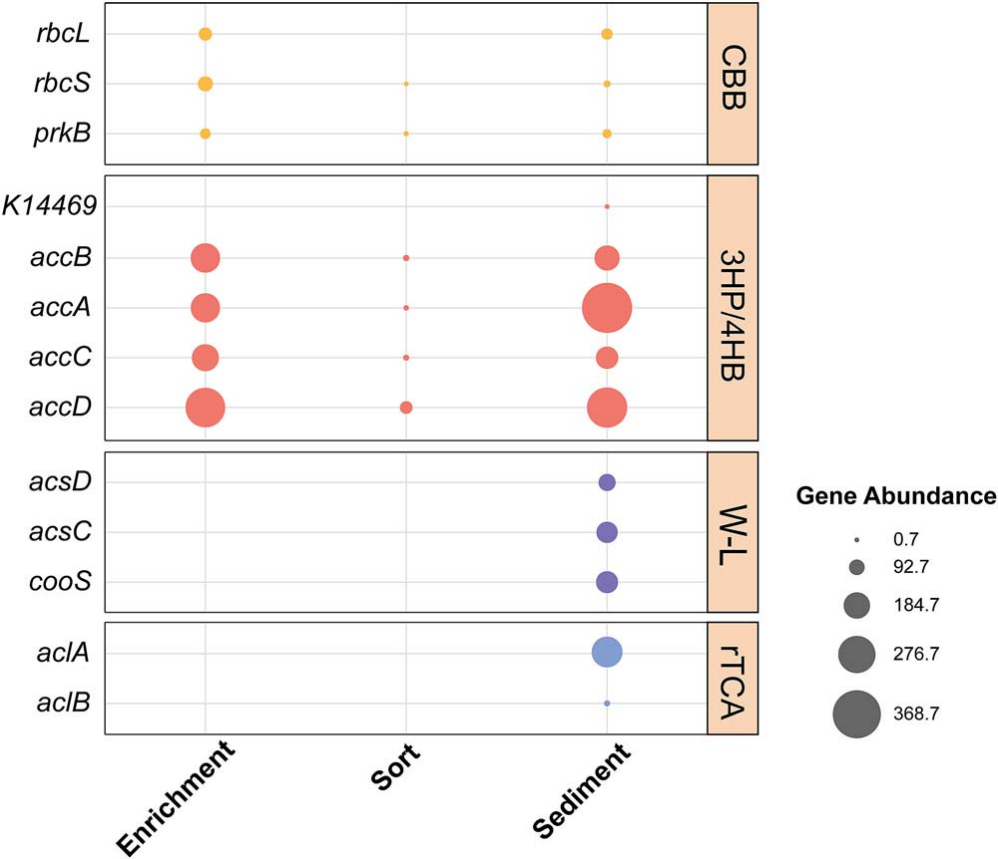
Relative abundances of key genes involved in major carbon fixation pathways across different samples, including the Calvin–Benson–Bassham (CBB) cycle, the 3-hydroxypropionate/4-hydroxybutyrate (3HP/4HB) cycle, the reductive tricarboxylic acid (rTCA) cycle, and the Wood–Ljungdahl (W–L) pathway, expressed as reads per kilobase per million mapped reads (RPKM).

**Figure 4:**
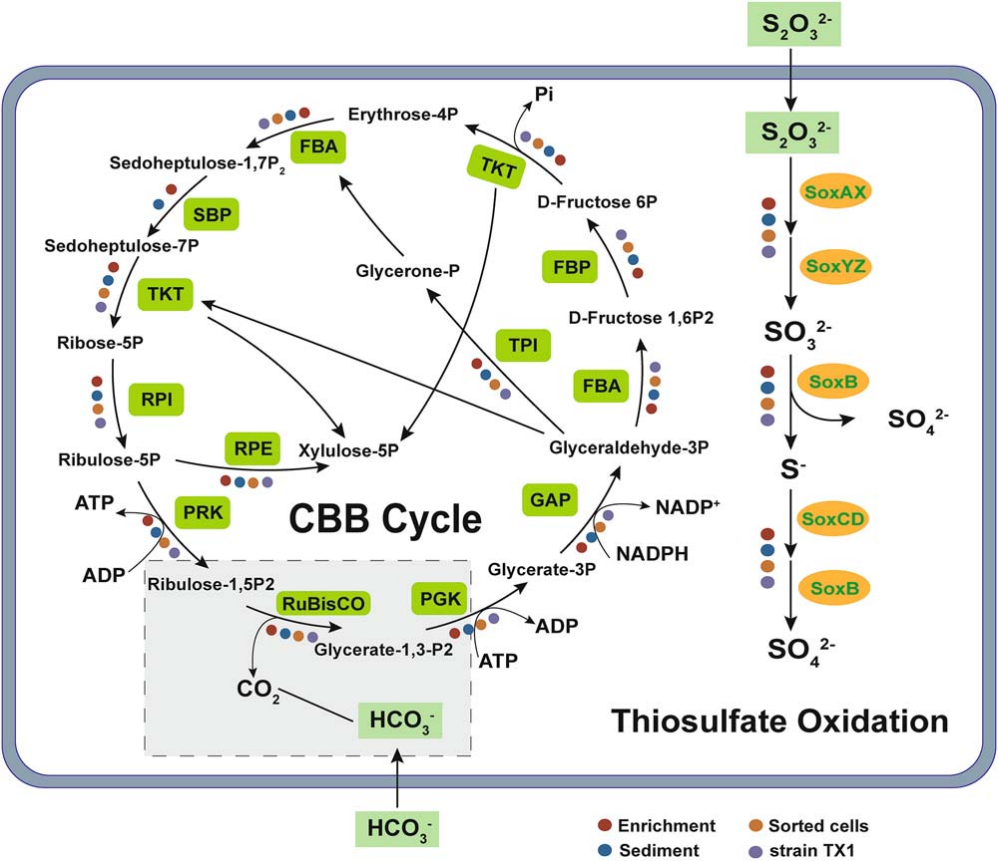
Metabolic potential scheme derived from metagenomic annotations of different samples. Red, blue, orange and purple points represent enrichment, sediment, sorted bacteria and strain TX1 samples, respectively.

Further analysis of the sequencing data focused on identifying metabolic pathways associated with carbon fixation function. Key carbon fixation gene relative abundance analysis indicated the presence of multiple key carbon fixation genes in the coastal sediment, particularly for the *accA* gene involved in the 3-hydroxypropionate/4-hydroxybutyrate (3HP/4HB) cycle, with the highest abundance of 386.0 reads per kilobase per million (RPKM). In addition to the CBB cycle and 3HP/4HB cycle, genes affiliated with the Wood-Ljungdahl (W-L) pathway, a well-recognized anaerobic carbon fixation route, were also included in the analysis to provide a comprehensive overview of potential carbon fixation strategies in coastal sediments. The enrichment and sorted cells showed similar key carbon fixation genes, which were both concentrated in the CBB cycle and the 3HP/4HB cycle, with a higher abundance of the *accABCD* gene from the 3HP/4HB cycle, reaching 200 RPKM in enrichment and over 10 RPKM in sorted cells. Additionally, both enrichment and sorted cells contained *rbcS* and *prkB* genes from the CBB cycle, with relative abundances of 87.41 and 50.49 RPKM, respectively, in enrichment, and 0.87 and 3.75 RPKM, respectively, in sorted cells, indicating that both sets of cells potentially fix carbon through the CBB cycle (Fig. 3; Table S2). To reveal the carbon fixation metabolic potential of sorted cells in the enrichment and the sediment, KEGG annotations were carried out to demonstrate that the enrichment and sediment MAGs both contained all the genes necessary for the CBB cycle, whereas the sorted cell genomes encoded all CBB cycle-related genes except for glpx-SEBP (Fig. 4). Through the reconstruction of genomic metabolic pathways, the presence of functional genes related to the carbon fixation pathway was successfully identified, confirming that SCRS can effectively identify and sort chemoautotrophic bacteria from environment.

### Isolation and characterization of a dominant thiosulfate-oxidizing chemoautotrophic strain in sorted cells

**Figure 5:**
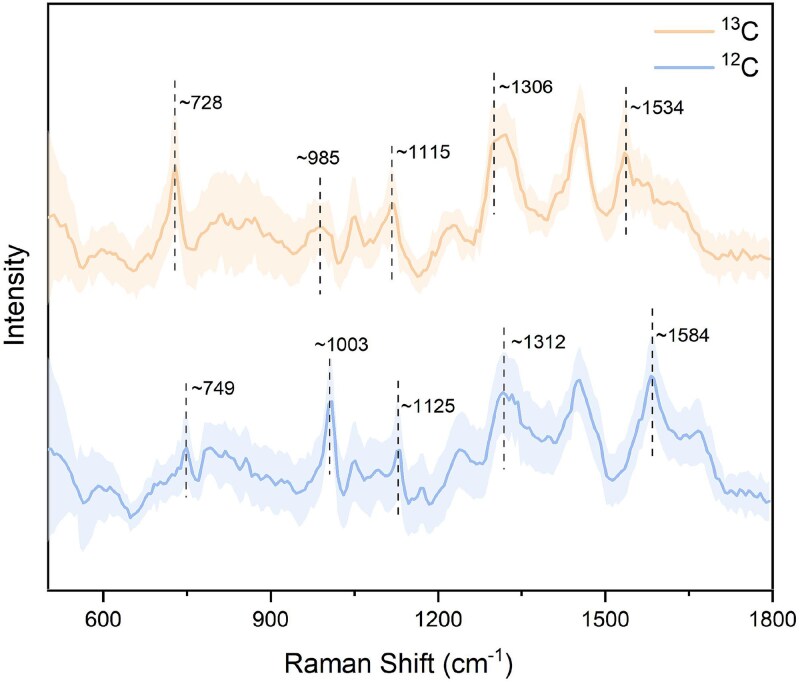
Mean Raman spectra of strain TX1 incubated 5 days with ^13^C-NaHCO_3_ (orange) and ^12^C-NaHCO_3_ (blue). Shaded areas represent the standard deviation of the mean spectra derived from multiple single-cell measurements.

Based on the enrichment, a thiosulfate-oxidizing chemoautotrophic strain, designated as TX1, was isolated and purified, and its morphology was observed by transmission electron microscopy (Fig. S3). Growth dynamics of strain TX1 under autotrophic conditions are shown in Fig. S4. When subjected to the ^13^C isotope labeling for 5 days and SCRS, the Raman spectra of strain TX1 under different carbon source conditions revealed that strain TX1 exhibited Cyt *c* Raman band shifts from 749, 1125, 1312, and 1584 cm^−1^ to 728, 1115, 1306, and 1534 cm^−1^, respectively, along with a phenylalanine band shift from 1003 cm^−1^ to 985 cm^−1^ (Fig. 5). The intermediate phenylalanine band shift indicates partial ^13^C substitution within the benzene ring, consistent with incomplete isotopic labeling. These shifts were consistent with those observed in the representative strain *Thiobacimonas profunda* CGMCC 1.12377^T^ and the enrichment samples, suggesting that strain TX1 could assimilate ^13^C for carbon fixation activity.

**Figure 6:**
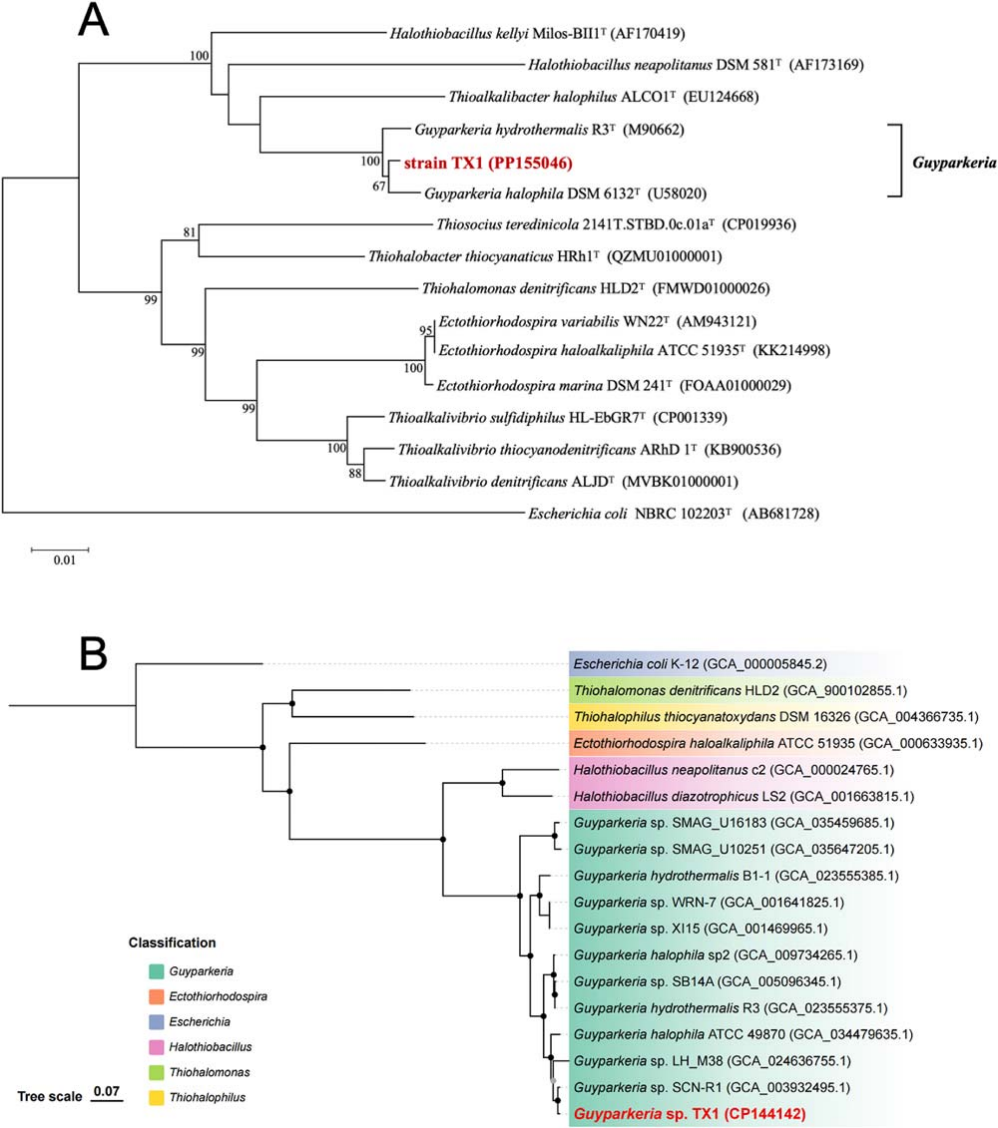
Phylogenetic positions of the carbon fixation isolates from sediment and their affiliated taxa based on the 16S rRNA gene (A). The genome tree of strain TX1 based on orthologous clusters (B). Black circles inside phylogenetic tree marked nodes with bootstrap values not lower than 90%. Grey circles marked nodes with bootstrap values lower than 90% but higher than 70%.

The phylogeny based on 16S rRNA gene sequences indicated that strain TX1 should be classified into the genus *Guyparkeria* (Fig. 6A), with the highest sequence identities with *Guyparkeria halophila* DSM 6132^T^ (99.2%). However, the ANI value between strain TX1 and *Guyparkeria halophila* DSM 6132^T^ was 88.7%, which was below the species delineation threshold of 95% as recommended by Chun et al. [53], proposing that strain TX1 represents a novel chemoautotrophic species. Phylogenetic analysis of the TX1 genome further confirmed its close relationship with other *Guyparkeria* strains (Fig. 6B), of which only two culturable species have been reported to date [54], demonstrating that strain TX1 represents a new culturable species in *Guyparkeria* genus.

The genus *Guyparkeria* has the ability to fix carbon through the CBB cycle, as shown by the results of Lau et al. [47]. To further elucidate the carbon fixation mechanism of strain TX1, the reconstructed metabolic pathways based on KEGG annotations found that strain TX1 genome harbored CBB cycle-related genes including *rbc* and also contained a complete metabolic pathway for thiosulfate oxidation (Fig. 4), indicating that strain TX1 had the potential for carbon fixation via the CBB cycle, with thiosulfate as the energy source (Fig. 4). To validate whether strain TX1 originated from the sorted-cell population and to assess its enrichment, metagenomic reads were mapped to the TX1 genome. In the sediment metagenome, TX1-related sequences accounted for ~0.012% of total reads. In contrast, in the sorted-cell metagenome, ~19.4% of reads could be mapped to the TX1 genome, covering 98.68% of the genome at an average sequencing depth of 651×. BLASTn analysis of assembled contigs from the sort-cell metagenome against the TX1 genome revealed multiple high-identity alignments (≥99% identity and ≥500 bp in length) distributed throughout the genome (Table S3). Furthermore, sequence comparison of carbon fixation-related genes and thiosulfate oxidation genes between strain TX1 and the sorted cells revealed a similarity of 100% (Table S4), these results strongly support that strain TX1 was present in the sorted-cell pool and was successfully enriched and isolated. The carbon-fixing genes of CBB cycle and thiosulfate oxidation genes annotated in the metagenomic assemblies were compared for sequence similarity against the UniProt database (Table S5).

### Carbon fixation capacity of strain TX1

Bacterial suspensions of strain TX1 was collected on days 0, 3, 5, 7, and 9 for TOC analysis, with the TOC value on day 0 serving as the background and subtracted from subsequent measurements. The bacterial suspensions were filtered using a 0.7 μm glass fiber membrane, with the organic carbon in the filtrate representing the dissolved organic carbon (DOC) concentration of strain TX1, while the organic carbon retained on the filter represented the particulate organic carbon (POC) concentration. The sum of DOC and POC was considered the TOC concentration of strain TX1.

**Figure 7:**
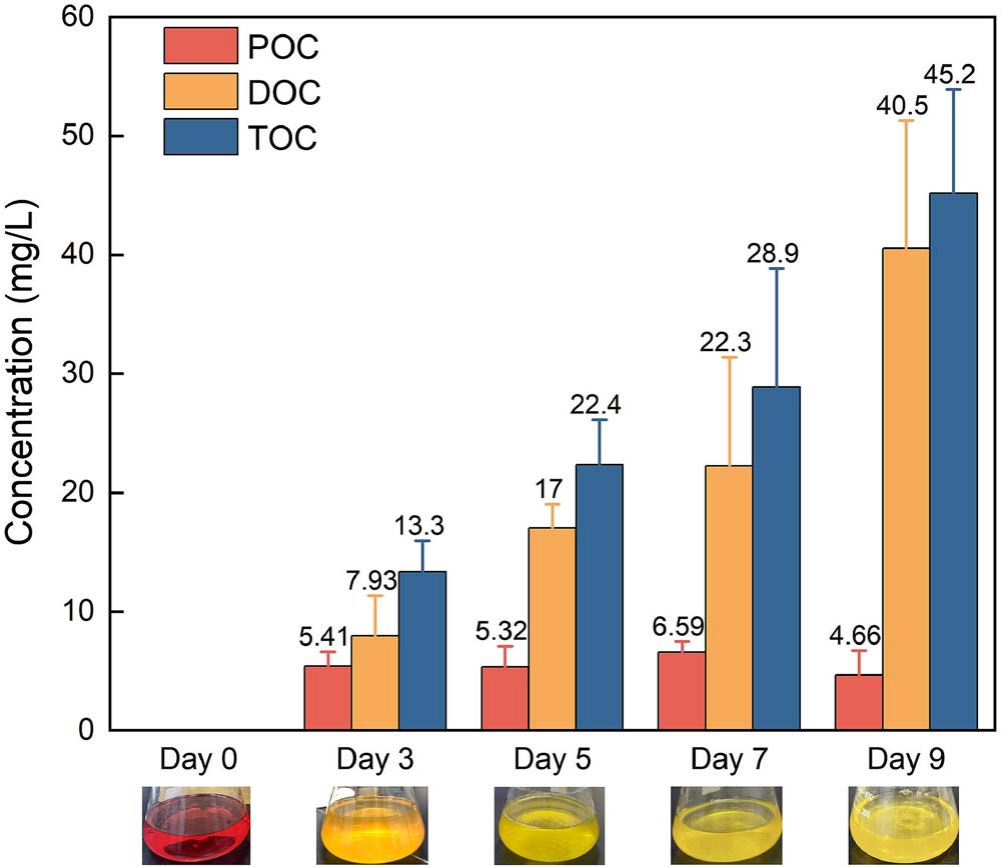
Organic carbon concentrations in strain TX1 cultures at days 0, 3, 5, 7, and 9 of cultivation. The visual appearance of strain TX1 cultures corresponding to each time point is shown below the timeline.

The POC concentration of strain TX1 increased significantly compared to day 0, reaching a peak of 6.59 mg/L on day 7 (Fig. 7). Similarly, the DOC concentration of strain TX1 also showed a significant increase from day 0 and continued to rise throughout the cultivation period, from 7.93 mg/L on day 3 to 40.5 mg/L on day 9. The TOC concentration of strain TX1, calculated as the sum of DOC and POC, exhibited a consistent upward trend, increasing from 13.3 mg/L on day 3 to 45.2 mg/L on day 9. These findings demonstrated the substantial carbon fixation capacity of strain TX1.

## Discussion

### Cyt *c* and phenylalanine as functional Raman biomarkers of chemoautotrophic activity

The Raman bands of Cyt *c* appeared at 749, 1129, 1312, and 1589 cm^−1^, corresponding to pyrrole breathing mode, C-N stretching, C-H bending, and C-C stretching vibrations, respectively [55–57]. Concurrently, phenylalanine displayed a Raman band near 1002 cm^−1^ [58]. As observed in SCRS, the assimilation of ^13^C-NaHCO_3_ by active carbon fixers induced distinct shifts in both Cyt *c* and phenylalanine Raman bands. These shifts arise from the isotopic effect of ^13^C incorporation, where the increased atomic mass of ^13^C reduces vibrational frequencies of carbon-containing molecules, resulting in detectable redshifts in Raman bands associated with Cyt *c* and phenylalanine.

Importantly, Cyt *c* and phenylalanine were not intended to serve as pathway-specific or taxonomic biomarkers of chemoautotrophy. Instead, their Raman band shifts were used as functional biomarkers of isotopic carbon incorporation and anabolic activity at the single-cell level. Any microorganism actively assimilating inorganic carbon into cellular biomass is expected to exhibit isotope-induced shifts in carbon-associated vibrational modes, regardless of its taxonomic identity.

The selection of these Raman features is mechanistically grounded. Cyt *c* is a heme protein ubiquitously present in bacteria, and plays dual roles in microbial energy metabolism. It is essential for ATP synthesis and serves as an electron carrier in respiratory chains [59]. For example, in green sulphur bacteria, Cyt *c* mediates electron transfer between sulfide oxidation and terminal electron acceptors [60]. Moreover, Cyt *c* displays maximum electronic absorption around 550 nm [61], which is compatible with the 532 nm laser used in this study. This resonance Raman effect enhances the Raman signal of Cyt *c* relative to other cellular components, such as the protein-related band at 1240 cm^−1^ and 1664 cm^−1^, and the lipid-related band at 1450 cm^−1^, facilitating the detection of isotope-induced spectral shifts. Consistently, genes encoding Cyt *c* were identified in enrichment MAGs and Raman-sorted cell contigs (Table S6), providing genomic support for the Cyt *c*-associated Raman signatures observed here.

The ring breathing vibration of phenylalanine, observed around 1002 cm^−1^, exhibits a strong band, making it one of the most reproducible spectral features in microbial Raman spectra [30, 62]. Its consistent intensity, low spectral interference, and predictable isotope-induced red shift have led to its widespread use as a reporter of anabolic activity and cellular growth in stable isotope probing Raman studies [30]. In contrast, other aromatic amino acids like tyrosine and tryptophan exhibit suppressed Raman signals due to differences in substituent symmetry and resonance behavior, rendering them less suitable as universal activity biomarkers [62].

The conjugated porphyrin ring structure of Cyt *c* and the benzene ring of phenylalanine make their vibrational modes particularly sensitive to carbon isotope substitution. This molecular sensitivity, combined with active carbon assimilation under the experimental conditions applied here, explains why these two Raman features consistently exhibited significant redshifts in cells identified as active carbon fixers. The concurrent shifts in Cyt *c* and phenylalanine provide a robust phenotypic signature of intracellular ^13^C incorporation and anabolic activity at the single-cell level. Crucially, this SIP-Raman framework enables non-destructive, real-time activity mapping of intracellular carbon assimilation within intact sediments at single-cell resolution. Unlike genomic methods requiring cell lysis or bulk isotopic assays, this approach directly links metabolic activity to individual cells, advancing quantification of microbial roles in carbon fixation.

### Sulfur-coupled carbon fixation in coastal sediment microbiomes

In this study, metagenomic analysis of coastal sediment enrichments recovered four medium- and high-quality MAGs. Functional annotation and reconstruction of carbon fixation pathways of these four MAGs revealed that most of them contained genes associated with the CBB cycle (Fig. S2). However, only Enrichment_bin1 encoded the key enzyme RuBisCO, indicating a complete genetic potential for autotrophic carbon fixation. Taxonomic classification using GTDB-Tk showed that Enrichment_bin1 belongs to the genus *Guyparkeria*, a well-established sulfur-oxidizing chemoautotrophic lineage known to fix carbon via the CBB cycle. In contrast, the other enrichment-derived MAGs were classified as non-autotrophic taxa, and accordingly lacked RuBisCO genes in their genomes. Subsequent sorting of cells exhibiting ^13^C-induced Raman biomarker shifts, followed by targeted genome sequencing, validated near-complete CBB pathways in these sorted active chemoautotrophs, demonstrating the utility of Raman-based phenotypic screening.

A novel chemoautotrophic strain, *Guyparkeria* sp. TX1, was isolated from the enrichments. SCRS and genome sequencing indicated that strain TX1 exhibited carbon fixation phenotypic traits and carried genes related to the CBB cycle. Sequencing reads mappings revealed that 19.4% and 23.7% of the reads from the genome of strain TX1 mapped to the target genome and the enrichment genome, respectively. Moreover, strain TX1 was classified within the same taxonomic group as bin1 from the enrichment sample, demonstrating that a cultured chemoautotrophic strain was successfully obtained through this targeted enrichment. Intriguingly, although the reconstructed carbon fixation pathway indicated that strain TX1 lacked sedoheptulose-1,7-bisphosphatase (SBPase) in the CBB cycle, the carbon fixation effect of strain TX1 was significant. However, one isoenzyme, namely fructose-1,6-bisphosphatase (FBPase), in autotrophic prokaryotes exhibits substrate broad-spectrum activity i.e. capable of hydrolyzing both FBP and SBP [63–65]. Based on the autotrophic conditions in this study, we hypothesize that the FBPase annotated in the sorted cells and strain TX1 is a bifunctional enzyme hydrolyzing both FBP and SBP. This metabolic plasticity ensures CBB cycle functionality under autotrophic sulfide-rich conditions, highlighting evolutionary adaptations to coastal sediment redox gradients.

Dark carbon fixation mediated by chemoautotrophic microorganisms has emerged as a significant carbon sink in coastal ecosystems [66], and the CBB cycle has been identified as the dominant carbon fixation pathway in coastal sediments in previous studies [7]. Due to the periodic exposure and submergence caused by tidal cycles, coastal sediments exhibit highly dynamic redox conditions. These redox processes are closely coupled with the sedimentary carbon, nitrogen, and sulfur cycles, providing abundant substrates for chemoautotrophic activity [67]. Among these, sulfate reduction accounts for ~50%–70% of total respiration in coastal sediments, generating substantial amounts of reduced inorganic sulfur compounds that serve as major energy sources driving chemoautotrophic carbon fixation [9]. Previous studies have observed a high abundance and transcriptional activity of sulfur oxidation genes in coastal sediments, suggesting that chemoautotrophic microorganisms commonly utilize reduced sulfur as an electron donor in these environments [14, 68, 69]. Coastal sediments typically harbor large populations of sulfur-oxidizing autotrophic bacteria [7, 70, 71], with *Gammaproteobacteria* being the most ubiquitous class, accounting for 70%–86% of dark carbon fixation in these environments [7, 72, 73]. The representative chemoautotrophic strain investigated in this study, which derives energy from sulfur oxidation and utilizes the CBB cycle for carbon fixation [34], exhibited carbon fixation phenotypic information that were clearly reflected in SCRS. Genome analysis of strain TX1 isolated in this study indicated that it belongs to the genus *Guyparkeria*, a member of class *Gammaproteobacteria*. Members of this genus have been reported to possess genes for both the CBB cycle and the thiosulfate oxidation pathway, making them typical sulfur-oxidizing autotrophic microorganisms [47]. Functional annotations of the coastal sediment, enrichments, sorted cells, and strain TX1 revealed the presence of complete pathways of both the CBB cycle and thiosulfate oxidation (Fig. 4), and all samples exhibited carbon fixation phenotypic information of SCRS. Notably, genes involved in sulfur oxidation were highly abundant across sediments, enrichments, and sorted cells (Table S2). In particular, the *soxB* gene reached a relative abundance of 29.6 RPKM in sediments, while *soxY* exhibited an exceptionally high abundance of 776.2 RPKM, other *sox* genes also showed abundances exceeding 200 RPKM. The coupling of dark carbon fixation with the CBB cycle and sulfur oxidation appears to be a widespread phenomenon in coastal sediments. Further investigations into the interactions between sulfur oxidation and the CBB cycle can help to understand the biogeochemical functions of coastal sediments and their responses to climate change more comprehensively.

## Conclusion

Altogether, this study establishes the application of Cyt *c* and phenylalanine Raman band shifts as single-cell functional biomarkers for detecting intracellular ^13^C assimilation in chemoautotrophic microorganisms through the integration of stable isotope probing and SCRS. Additionally, a dominant chemoautotroph *Guyparkeria* sp. TX1 in the enrichment was successfully isolated, it was consistent with the groups obtained by targeted sorting, and its carbon fixation efficiency was detected. These findings advance both methodological and mechanistic understanding of microbial ecology. The Raman-based approach bridges single-cell metabolic activity with community-scale carbon fluxes, overcoming limitations of bulk omics methods, while the elucidation of sulfur-coupled CBB cycling establishes its critical role in coastal blue carbon sinks. This study provides mechanistic insights for modeling carbon storage dynamics in coastal ecosystems and informs targeted strategies for coastal carbon management, demonstrating the potential of microbial activity mapping to refine carbon flux quantifications in environments.

## Supplementary Material

ycag073_Supplementary_materials

## Data Availability

All metagenomic sequences are available under NCBI Bioproject PRJNA1205351. The 16S rRNA and genome sequences of strain *Guyparkeria* sp. TX1 are deposited in GenBank under accession numbers of PP155046 and CP144142, respectively.
