# Supplementary material for: 13C-labeled single-cell Raman sorting reveals sulfur-driven dark carbon fixation in coastal sediments
Source: ISME Commun. 2026 Mar 24;6(1):ycag073. doi: 10.1093/ismeco/ycag073 (PMC13082230; doi:10.1093/ismeco/ycag073)
Supplement: ycag073_Supplementary_materials [file ycag073_supplementary_materials.zip › Supplementary_materials_ycag073.docx]

**Supplemental Information**

**SI 1. Nycodenz density gradient separation protocol**

0.5 g of sediment was homogenized in 5 mL of phosphate-buffered saline (PBS; NaCl 8 g/L, KCl 0.2 g/L, Na_2_HPO_4_ 1.44 g/L, KH_2_PO_4_ 0.24 g/L), supplemented with 0.5% (v/v) Tween 20 (Aladdin), and then vortexed vigorously for 30 minutes at room temperature to obtain a sediment suspension. A solution of Nycodenz (≥98%, Aladdin) with a density of 1.42 g/mL (80%, w/v) was prepared, and the as-prepared slurry was slowly added to an Eppendorf tube containing 5 mL of the Nycodenz solution. The tube was centrifuged at 14000 g for 30 minutes at 4 °C. The middle layer containing microorganisms was collected and washed three times with ultrapure water to remove residual PBS and other trace reagents.

**SI 2. Bioinformatic workflows and software parameters of sorted cells** **and sediment samples**

Raw reads of sorted cells were filtered and trimmed using Trimmomatic version 0.39 [1] to generate clean reads. SPAdes version 3.10.1 [2] was used to assemble the clean reads with the parameter “-sc”. Gene prediction was conducted using Prokka version 1.14.5 [3], and functional orthologous genes annotation of protein-coding genes was performed using eggNOG-mapper version 2.1.4 [4]. The abundances of contigs were calculated using BWA version 0.7.17 [5], SAMtools version 1.9 [6], and BEDTools version 2.26.0 [7].

Metagenomic sequencing of sediment samples and enriched bacterial cultures was performed at Magigene Technology Corporation (Guangdong, PR China). After data acquisition, sequences were assembled using the assembly module of MetaWRAP software version 1.2.1[8], and contigs ≥300 bp were retained. The assembled contigs were preliminarily binned using MetaBAT 2 [9], MaxBin 2.0 [10] and CONCOCT [11]. The resulting bin sets were subsequently consolidated using the bin_refinement module. The quality of metagenome-assembled genomes (MAGs) was assessed using CheckM software version 1.1.3 [12], and the high- and medium-quality MAGs (completeness higher than 50% and contamination lower than 10%) were retained. Functional gene annotation followed the same methods as for targeted genomic sequencing. Taxonomic classification of MAGs was verified, and a phylogenetic tree was constructed using the GTDB-Tk software version 1.6.0 [13].

**SI 3. Bioinformatic workflows of carbon-fixing strain**

Genomic DNA was extracted from pure cultures using the Magnetic Soil and Stool DNA Kit (TIANGEN Biotech, Beijing, China) according to the manufacturer’s instructions and sent to Magigene Technology Corporation for whole-genome sequencing, with the genomic assembly and quality estimation performed as described previously. Targeted metagenomic reads were mapped to the genome of strain TX1 using Bowtie2 software version 2.5.1 [14]. The resulting SAM files were converted to BAM files with SAMtools version 1.9 [6] to calculate coverage. BLASTn version 2.15.0 [15] was used to align assembled contigs from the single-cell metagenome to the genome of strain TX1, and hits with ≥ 99% nucleotide identity and alignment lengths of ≥ 500 bp were retained for further analysis. Average nucleotide identity (ANI) was calculated using FastANI [16] to assess genomic similarity. A maximum-likelihood phylogenomic tree was constructed using IQ-Tree version 1.6.12 with the parameter “-st AA -bb 1000”, and the best amino acid substitution model was estimated using the same software [17].

**SI 4. Composition of culture media**

Chemical composition of carbon-free isolation medium:

NaCl (20.0 g/L), Na_2_S_2_O_3_ (5.0 g/L), NaHCO_3_ (2 g/L), KH_2_PO_4_ (1.8 g/L), Na_2_HPO_4_ (1.0 g/L), NH_4_Cl (0.5 g/L), MgSO_4_ (0.4 g/L), CaCl_2_ (0.05 g/L), FeCl_3_ (0.02 g/L) and MnSO_4_ (0.02 g/L), and the pH was adjusted to 7.5.

Chemical composition of DSMZ medium 574:

25 g NaCl, 2.5 g Na_2_S_2_O_3_·5H_2_O, 1 g (NH_4_)_2_SO_4_, 1.5 g MgSO_4_·7H_2_O, 0.4 g KH_2_PO_4_, 0.2 g NaHCO_3_, 0.3 g CaCl_2_·2H_2_O, 2 mL phenol red (0.5% w/v), 200 mL Tris-HCl buffer (0.1 M, pH 7.5), 800 mL sterile distilled water and 1 mL of trace element solution (per L of sterile distilled water: 50 g Na_2_-EDTA, 2.2 g ZnSO_4_·7H_2_O, 5.5 g CaCl_2_·2H_2_O, 5.1 g MnCl_2_·4H_2_O, 5 g FeSO_4_·7H_2_O, 1.1 g (NH_4_)_6_Mo_7_O_24_·4H_2_O, 1.6g CuSO_4_·5H_2_O, 1.6 g CoCl_2_·6H_2_O, adjusted to pH 7.0 using 2 M KOH), adjusted to pH 7.5. The medium was sterilized by filtration.

**References:**

1. Bolger AM, Lohse M, Usadel B. Trimmomatic: a flexible trimmer for Illumina sequence data. *Bioinformatics*. 2014;**30**:2114-20

2. Bankevich A, Nurk S, Antipov D *et al.* SPAdes: a new genome assembly algorithm and its applications to single-cell sequencing. *J Comput Biol*. 2012;**19**:455-77

3. Seemann T. Prokka: rapid prokaryotic genome annotation. *Bioinformatics*. 2014;**30**:2068-9

4. Cantalapiedra CP, Hernandez-Plaza A, Letunic I *et al.* eggNOG-mapper v2: functional annotation, orthology assignments, and domain prediction at the metagenomic scale. *Mol Biol Evol*. 2021;**38**:5825-29

5. Li H. Aligning sequence reads, clone sequences and assembly contigs with BWA-MEM. *arXiv*. 2013

6. Li H, Handsaker B, Wysoker A *et al.* The sequence alignment/map format and SAMtools. *Bioinformatics*. 2009;**25**:2078-79

7. Quinlan AR, Hall IM. BEDTools: a flexible suite of utilities for comparing genomic features. *Bioinformatics*. 2010;**26**:841-42

8. Uritskiy GV, DiRuggiero J, Taylor J. MetaWRAP-a flexible pipeline for genome-resolved metagenomic data analysis. *Microbiome*. 2018;**6**:158

9. Kang DD, Li F, Kirton E *et al.* MetaBAT 2: an adaptive binning algorithm for robust and efficient genome reconstruction from metagenome assemblies. *PeerJ*. 2019;**7**:e7359

10. Wu YW, Simmons BA, Singer SW. MaxBin 2.0: an automated binning algorithm to recover genomes from multiple metagenomic datasets. *Bioinformatics*. 2016;**32**:605-7

11. Beaulaurier J, Zhu S, Deikus G *et al.* Metagenomic binning and association of plasmids with bacterial host genomes using DNA methylation. *Nat Biotechnol*. 2018;**36**:61-69

12. Parks DH, Imelfort M, Skennerton CT *et al.* CheckM: assessing the quality of microbial genomes recovered from isolates, single cells, and metagenomes. *Genome Res*. 2015;**25**:1043-55

13. Chaumeil PA, Mussig AJ, Hugenholtz P *et al.* GTDB-Tk: a toolkit to classify genomes with the Genome Taxonomy Database. *Bioinformatics*. 2019;**36**:1925–27

14. Langdon WB. Performance of genetic programming optimised Bowtie2 on genome comparison and analytic testing (GCAT) benchmarks. *BioData Min*. 2015;**8**:1-7

15. Camacho C, Coulouris G, Avagyan V *et al.* BLAST+: architecture and applications. *BMC Bioinf*. 2009;**10**:1-9

16. Jain C, Rodriguez-R LM, Phillippy AM *et al.* High throughput ANI analysis of 90K prokaryotic genomes reveals clear species boundaries. *Nat Commun*. 2018;**9**:5114

17. Nguyen LT, Schmidt HA, von Haeseler A *et al.* IQ-TREE: a fast and effective stochastic algorithm for estimating maximum-likelihood phylogenies. *Mol Biol Evol*. 2015;**32**:268-74

**Fig. S1.** Raman-guided single-cell sorting of ^13^C-labeled metabolically active cells using Raman-activated cell ejection (RACE)

**Fig. S2**. Reconstructed ‘carbon fixation by calvin cycle’ pathway in KEGG by 4 MAGs of enrichment sample.

**Fig. S3.** Morphology of strain TX1 under transmission electron microscope.

**Fig. S4.** Growth dynamics of *Guyparkeria* sp. TX1 under autotrophic conditions. Changes in optical density (OD600) was monitored during incubation in organic carbon-free medium.

**Table S1.** Genomic characteristics of strain TX1 and 23 mid-high-quality MAGs.

**Table S2.** The relative abundances of key genes in the carbon-fixing and sulfur oxidation pathways annotated from different samples [unit: reads per kilobase per million (RPKM)].

**Table S3.** Summary of genomic consistency between the targeted metagenomic dataset of sorted cells and strain TX1.

**Table S4.** BLAST results of carbon-fixing genes of CBB cycle and thiosulfate oxidation genes annotated between strain TX1 and sorted cells.

**Table S5.** BLAST results of carbon-fixing genes of CBB cycle and thiosulfate oxidation genes annotated in the metagenomic assemblies against the UniProt database.

**Table S6.** Annotation of cytochrome *c*-related genes identified in enrichment cultures and Raman-sorted cells based on eggNOG-mapper.
