# Supplementary figures and images for: 13C-labeled single-cell Raman sorting reveals sulfur-driven dark carbon fixation in coastal sediments
Source: ISME Commun. 2026 Mar 24;6(1):ycag073. doi: 10.1093/ismeco/ycag073 (PMC13082230; doi:10.1093/ismeco/ycag073)

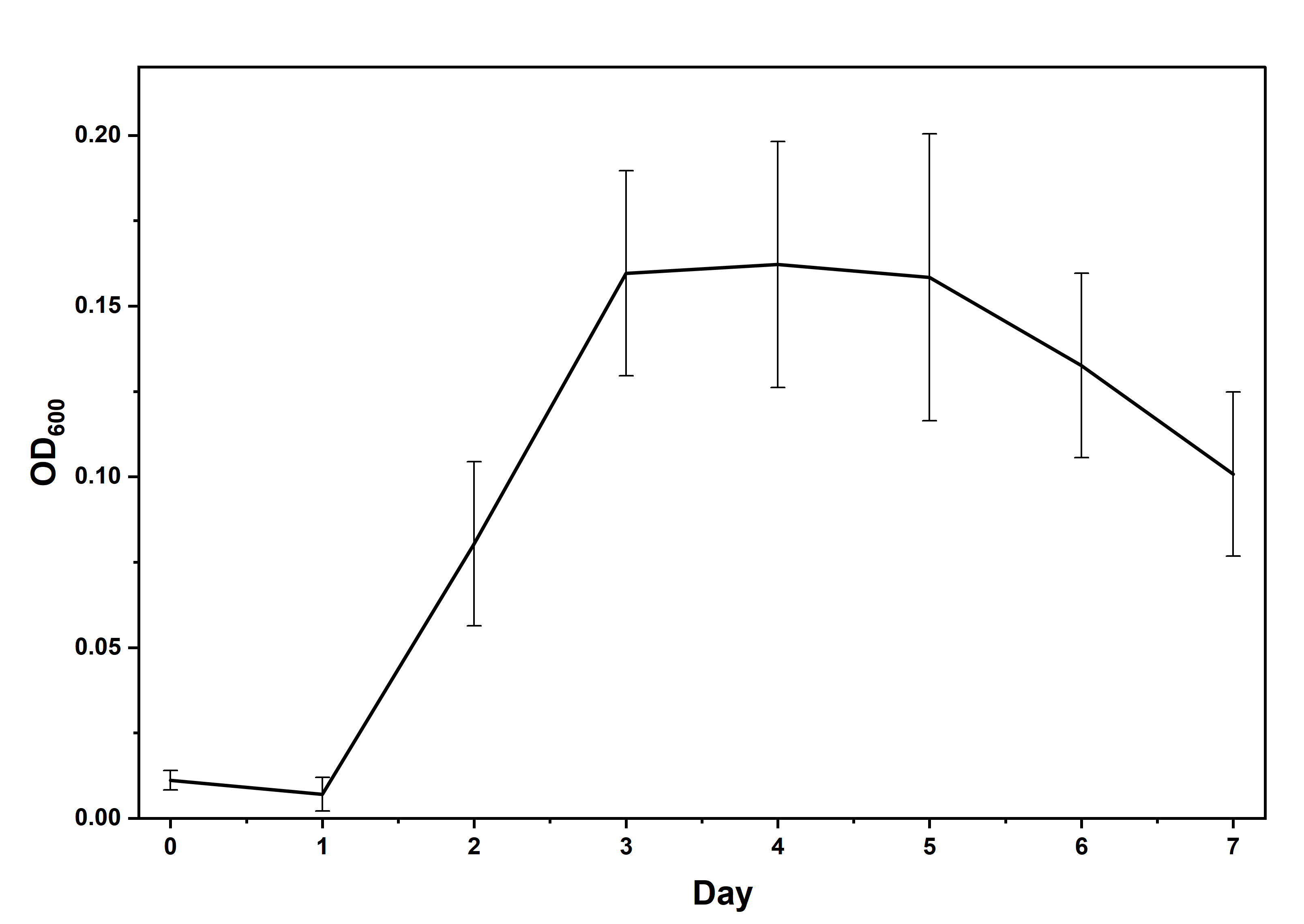

Supplement: ycag073_Supplementary_materials [file ycag073_supplementary_materials.zip › Supplementary_materials_ycag073_fig S4.tif]

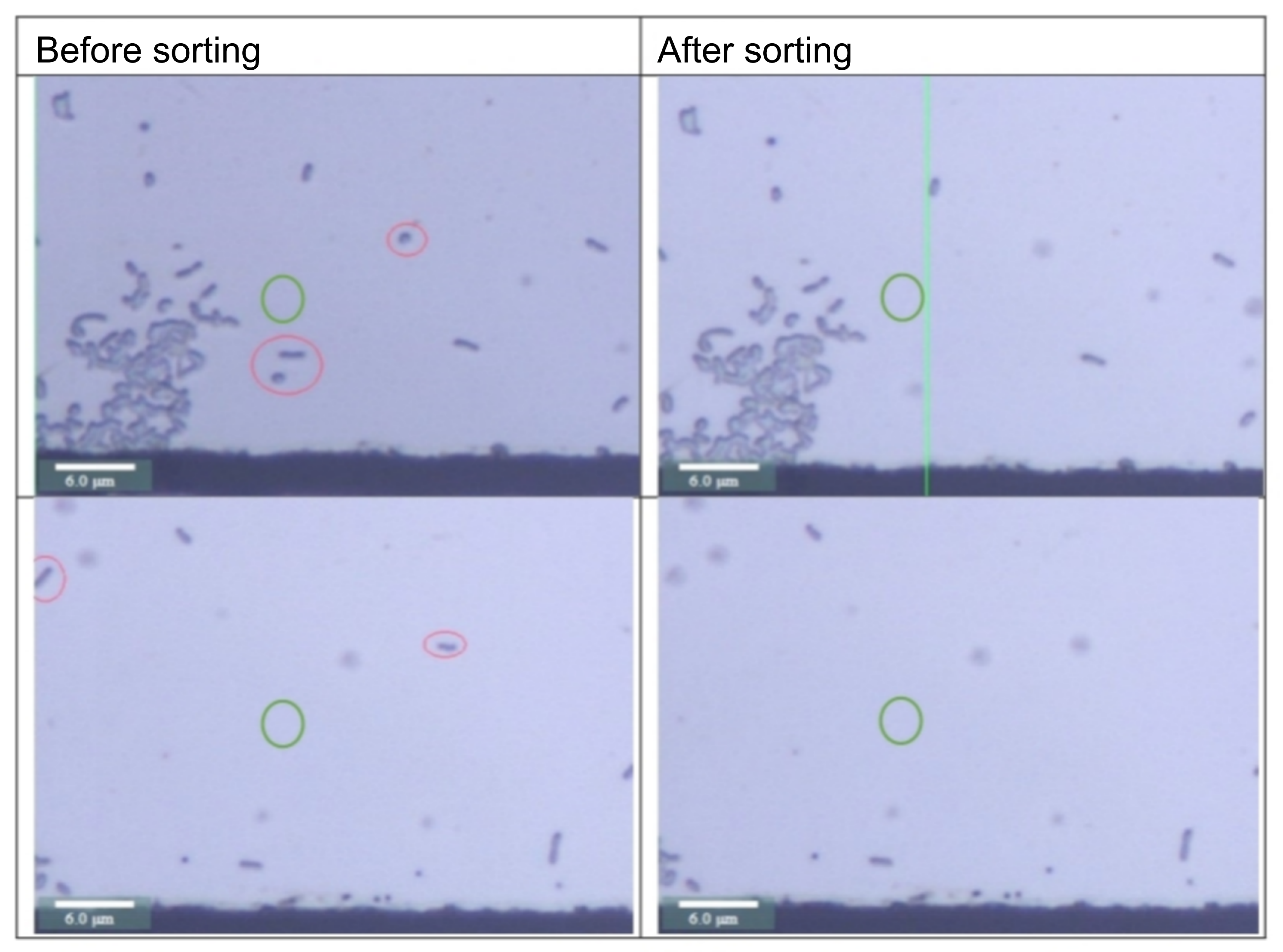

Supplement: ycag073_Supplementary_materials [file ycag073_supplementary_materials.zip › Supplementary_materials_ycag073_Figure S1.tif]

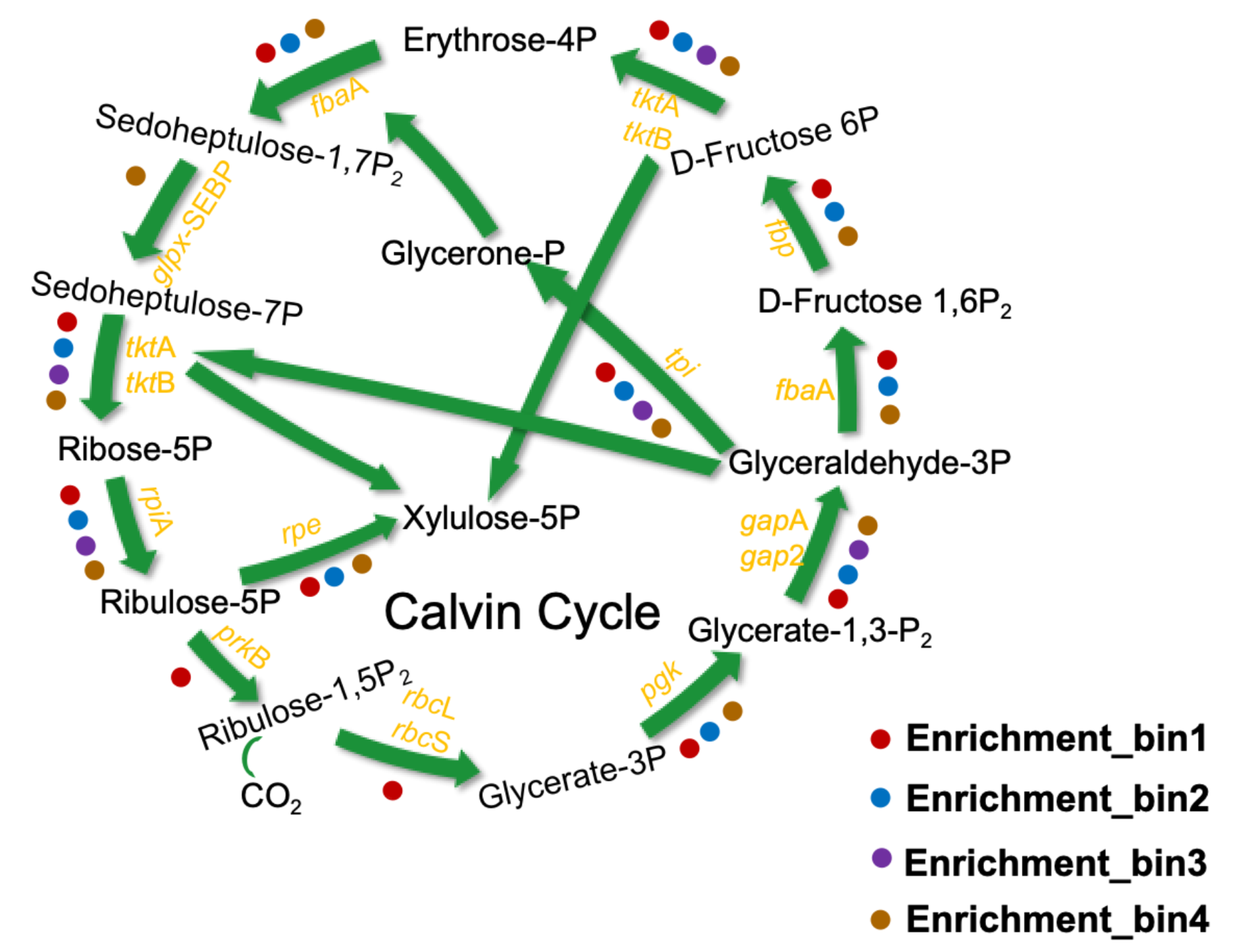

Supplement: ycag073_Supplementary_materials [file ycag073_supplementary_materials.zip › Supplementary_materials_ycag073_Figure S2.tif]

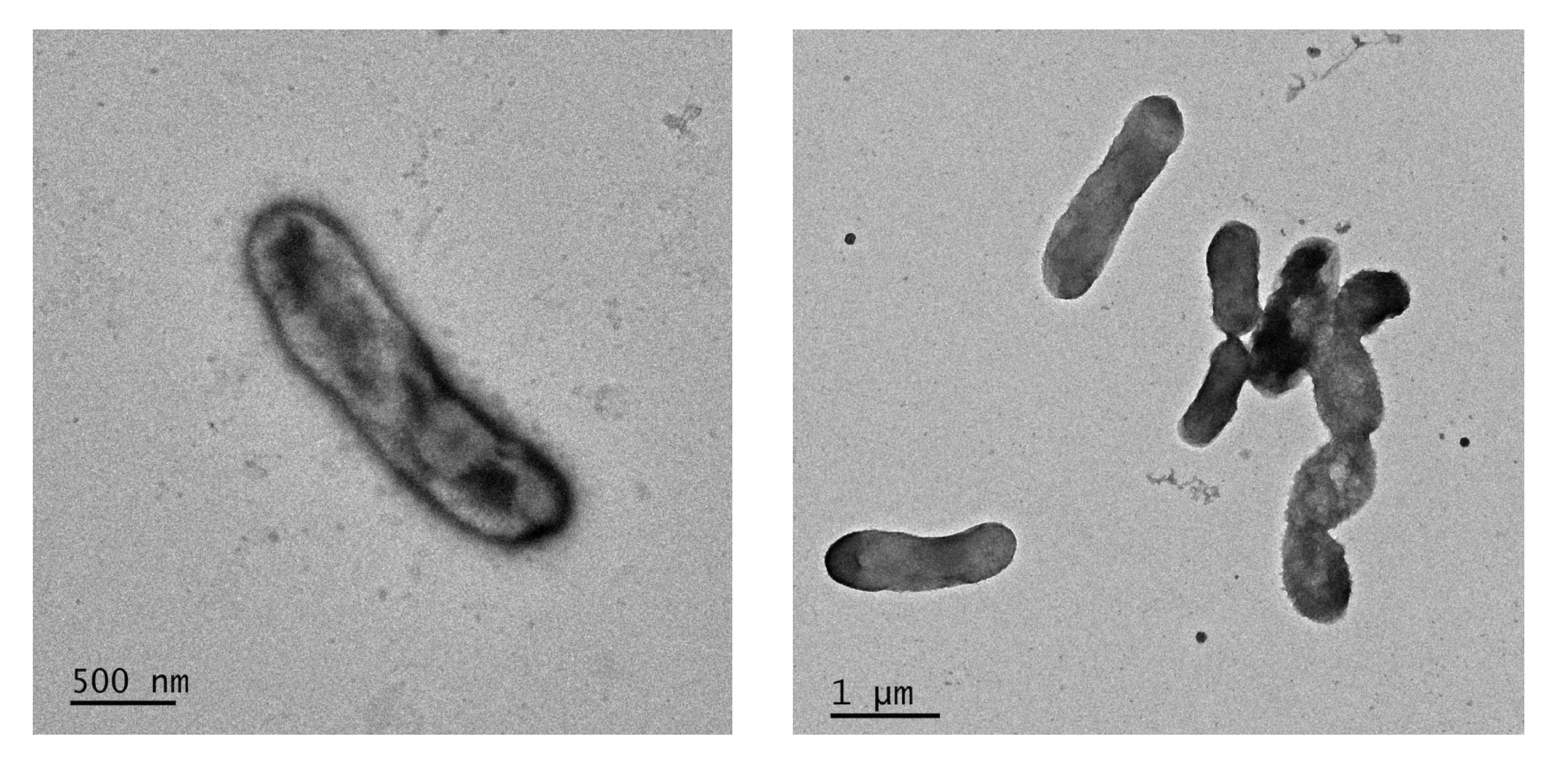

Supplement: ycag073_Supplementary_materials [file ycag073_supplementary_materials.zip › Supplementary_materials_ycag073_Figure S3.tif]
